# Macrophages communicate with mesangial cells through the CXCL12/DPP4 axis in lupus nephritis pathogenesis

**DOI:** 10.1038/s41419-024-06708-4

**Published:** 2024-05-18

**Authors:** Weiwei Li, Chun Yao, Haixia Guo, Xi’an Ni, Ran Zhu, Yongjun Wang, Bin Yu, Xuebing Feng, Zhifeng Gu, Zhanyun Da

**Affiliations:** 1 Department of Rheumatology, Affiliated Hospital of Nantong University, Medical School of Nantong University, Nantong, Jiangsu China; 2 Key Laboratory of Neuroregeneration of Jiangsu and Ministry of Education; Co-innovation Center of Neuroregeneration, Nantong University, Nantong, Jiangsu China; 3 Department of Rheumatology and Immunology, The Affiliated Drum Tower Hospital of Nanjing University Medical School, Nanjing, Jiangsu China

**Keywords:** Lupus nephritis, Lupus nephritis

## Abstract

Lupus nephritis (LN) occurs in 50% of cases of systemic lupus erythematosus (SLE) and is one of the most serious complications that can occur during lupus progression. Mesangial cells (MCs) are intrinsic cells in the kidney that can regulate capillary blood flow, phagocytose apoptotic cells, and secrete vasoactive substances and growth factors. Previous studies have shown that various types of inflammatory cells can activate MCs for hyperproliferation, leading to disruption of the filtration barrier and impairment of renal function in LN. Here, we characterized the heterogeneity of kidney cells of LN mice by single-nucleus RNA sequencing (snRNA-seq) and revealed the interaction between macrophages and MCs through the CXC motif chemokine ligand 12 (CXCL12)/dipeptidyl peptidase 4 (DPP4) axis. In culture, macrophages modulated the proliferation and migration of MCs through this ligand–receptor interaction. In LN mice, treatment with linagliptin, a DPP4 inhibitor, effectively inhibited MC proliferation and reduced urinary protein levels. Together, our findings indicated that targeting the CXCL12/DPP4 axis with linagliptin treatment may serve as a novel strategy for the treatment of LN via the CXCL12/DPP4 axis.

## Introduction

Systemic lupus erythematosus (SLE) is a complex systemic autoimmune disease that often affects kidney function [1]. Lupus nephritis (LN) is the most common cause of renal injury in SLE; it may manifest as asymptomatic proteinuria and hematuria or as hypertension, nephrotic syndrome, and acute nephritic syndrome. Approximately 10–30% of LN patients will progress to renal failure [2]. At present, LN is an important cause of morbidity and mortality in SLE patients. Though the pathogenesis mechanism of LN is still unclear, most scholars believe that it is caused by both genetic and environmental factors [3–5]. The production of autoantibodies and the deposition of immune complex (IC) in the renal parenchyma induce inflammatory cell infiltration and an immune imbalance, thereby causing renal injury and LN [6, 7]. The main drugs for treating LN include glucocorticoids, immunosuppressive agents, and biological agents [8, 9]. However, those treatments can also result in several problems, such as unsatisfactory complete remission rates, osteoporosis, infection, and obesity [10, 11]. Such poor outcomes pose a great challenge to current treatment strategies and necessitate the exploration of new therapeutic targets.

A variety of intrinsic cells in renal tissues are involved in LN progression [12]. Epithelial cell proliferation and inflammatory cell infiltration lead to crescent formation. Then, podocyte injury and fusion disappearance exacerbate proteinuria [13]. The hyperproliferation of mesangial cells (MCs) and stromal dilation result in a decrease in the filtration rate [14]. In addition, disturbance of the vascular endothelium promotes fibrosis of the arterial lining and monocyte and lymphocyte infiltration in tubular areas, contributing to interstitial tubulointerstitial pathologies [15, 16].

MCs account for 30–40% of the total number of glomerular cells. In addition to forming filter membranes, MCs can also secrete cytokines, growth factors, and extracellular matrix and can phagocytose macromolecules and apoptotic cells to restore glomerular stability [17–19]. However, in LN, the excessive proliferation of MCs and stroma dilation will block Baumann’s capsule, destroy the filtration barrier, and lead to renal fibrosis, ultimately resulting in glomerulosclerosis and renal failure [20, 21]. Thus, MCs are critical to glomerular homeostasis and the glomerular response to injury.

Immune imbalance, which can include imbalances in both innate and adaptive immunity, is the basis of LN [22, 23]. During this process, autoantibodies, such as anti-dsDNA and anti-nuclear antibodies, are produced. IC are deposited on endothelial cells and MCs. Podocyte autophagy is attenuated, and complement activation aggravates inflammatory responses [15, 24]. Activated immune cells produce various types of inflammatory mediators, including platelet-derived growth factor (PDGF), interleukin-6 (IL-6), interleukin-1 (IL-1), CXC motif chemokine ligand 13 (CXCL13), and interferon-γ (IFN-γ), which directly damage MCs and promote excessive proliferation. Moreover, studies have found that lymphangiogenesis is increased in LN patients, which may provide a “green channel” for macrophages and other cells [25]. However, in LN, the regulatory mechanism of macrophages on glomerular MCs is still not clear.

In this paper, we performed single-nucleus RNA sequencing (snRNA-seq) to define the cellular heterogeneity in the kidneys of LN mice and found that macrophages can regulate the proliferation and migration of MCs through the CXC motif chemokine ligand 12 (CXCL12)/dipeptidyl peptidase 4 (DPP4) axis. We then further verified the interactions between macrophages and MCs in vitro and confirmed that linagliptin, a DPP4 inhibitor, can inhibit the CXCL12/DPP4 axis to modulate MCs and decelerate LN progression.

## Results

### Biochemical and pathological alterations of LN mice

**Fig. 1:**
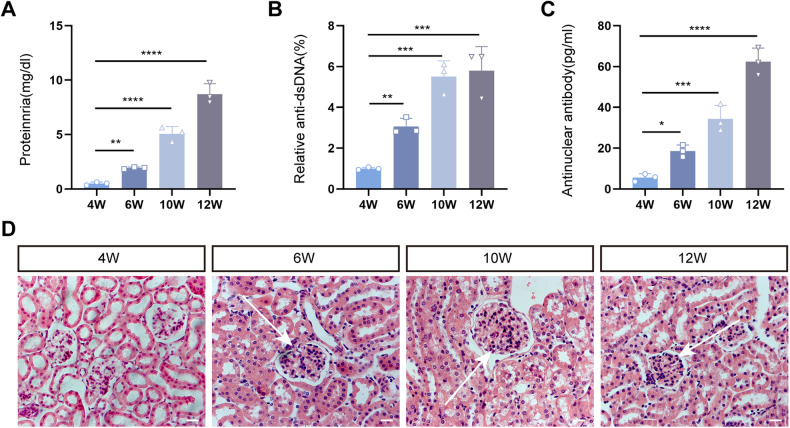
Biochemical and pathological alterations of LN mice. **A**–**C** The 24-h urine protein concentrations, and relative anti-dsDNA and anti-nuclear antibody levels in LN mice at 4, 6, 10, and 12 weeks. **D** HE staining of kidneys at different weeks. Scale bar = 20 μm, *n* = 3 for each group.

LN is usually accompanied by elevated anti-dsDNA antibodies and anti-nuclear antibodies (ANAs) as well as signs of renal insufficiency [26–28]. Therefore, we detected urinary protein concentrations and the above two serum indicators at 4, 6, 10, and 12 weeks in LN mice. The results showed that the 24-h urinary protein concentrations and serum anti-dsDNA antibody and ANA levels in LN mice increased weekly (Fig. 1A–C). Notably, the values at 6, 10, and 12 weeks were statistically different from those at 4 weeks. Moreover, renal hematoxylin–eosin (HE) staining revealed no lesion status in LN mice at 4 weeks, whereas the proliferation of glomerular MCs and vascular endothelial cells were obvious at 6, 10, and 12 weeks, and inflammatory cell infiltration gradually increased (Fig. 1D). We then selected kidneys from LN mice at 6 and 10 weeks for snRNA-seq analysis to explore the early stage of LN and to intervene at the early stage of the disease, delaying the occurrence and development of the disease.

### Identification of kidney cellular heterogeneity in LN mice by snRNA-seq

**Fig. 2:**
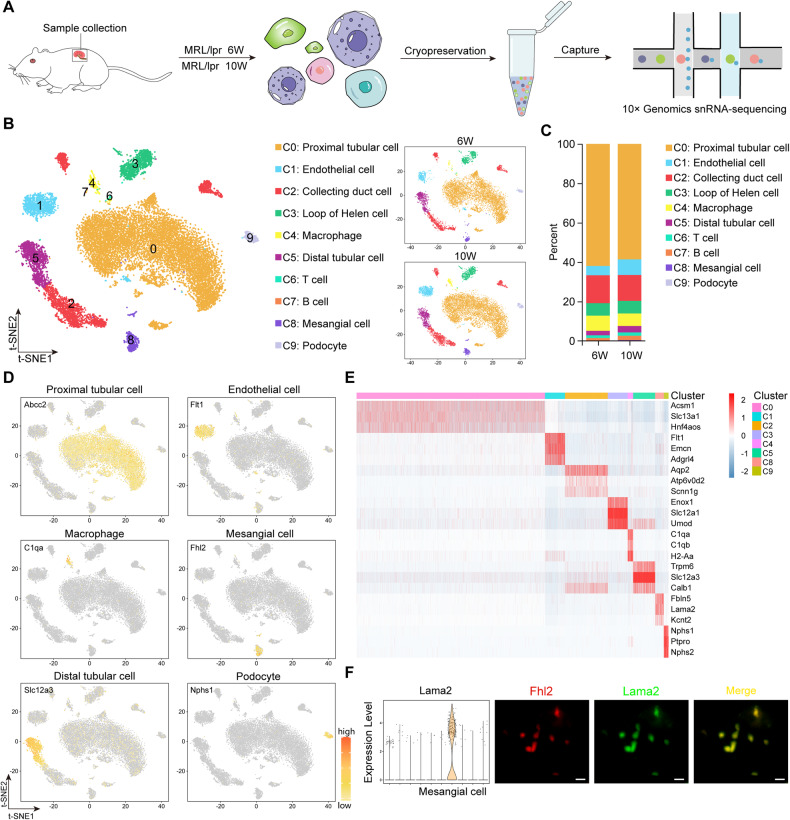
snRNA-seq landscape of kidneys in LN mice. **A** Schematic workflow for 10X Genomics snRNA-seq. **B** t-SNE analysis (left) showed that there were 10 different cell clusters in the renal cortex. The distributions of renal cells at 6 and 10 weeks are shown on the right side. Different cell clusters are color-coded. **C** Changes in the proportions of each cell cluster from 6 to 10 weeks. **D** t-SNE map showing the expression levels of single marker genes in proximal tubules, endothelial cells, macrophages, MCs, distal tubules, and podocytes. **E** Expression heatmap of the top three DEGs in the indicated cell clusters. **F** The expression of Lama2 on MCs was verified by co-immunolocalization staining with Fhl2. Scale bar = 5 μm, *n* = 3 for each group.

To explore the cellular composition and changes in the kidneys of LN mice at 6 and 10 weeks, we performed 10X Genomics snRNA-seq (Fig. 2A). We obtained transcriptome data from 17,188 cells, which were analyzed by cluster-free analysis and visualized by the t-Distributed Stochastic Neighbor Embedding (t-SNE) dimensionality reduction algorithm. According to the recently published single-cell data of mouse glomerular canonical markers [29], these cell groups were categorized into 10 clusters, namely, proximal tubules (C0), endothelial cells (C1), collecting ducts (C2), Helen’s rings (C3), macrophages (C4), distal tubules (C5), T cells (C6), B cells (C7), MCs (C8), and podocytes (C9) (Fig. 2B). The numbers of macrophages and glomerular MCs were elevated in LN mice from 6 to 10 weeks, consistent with the findings of the current study (Fig. 2C) [30]. Figure 2D shows the t-SNE mapping of classical marker genes in major cell clusters. An expression heatmap of specific differentially expressed genes (DEGs) in different cell types was then constructed (Fig. 2E). Notably, in addition to the classical MC markers, the gene laminin subunit alpha 2 (Lama2) was specifically expressed in MCs, indicating that it might be a new MC marker. We then performed immunostaining of four and a half LIM domains 2 (Fh12) and Lama2 on kidney sections of LN mice at 10 weeks, which confirmed that Lama2 might be a potential candidate marker for MCs (Fig. 2F). These findings are also consistent with a previous report [31].

### Sub-clustering of MCs

**Fig. 3:**
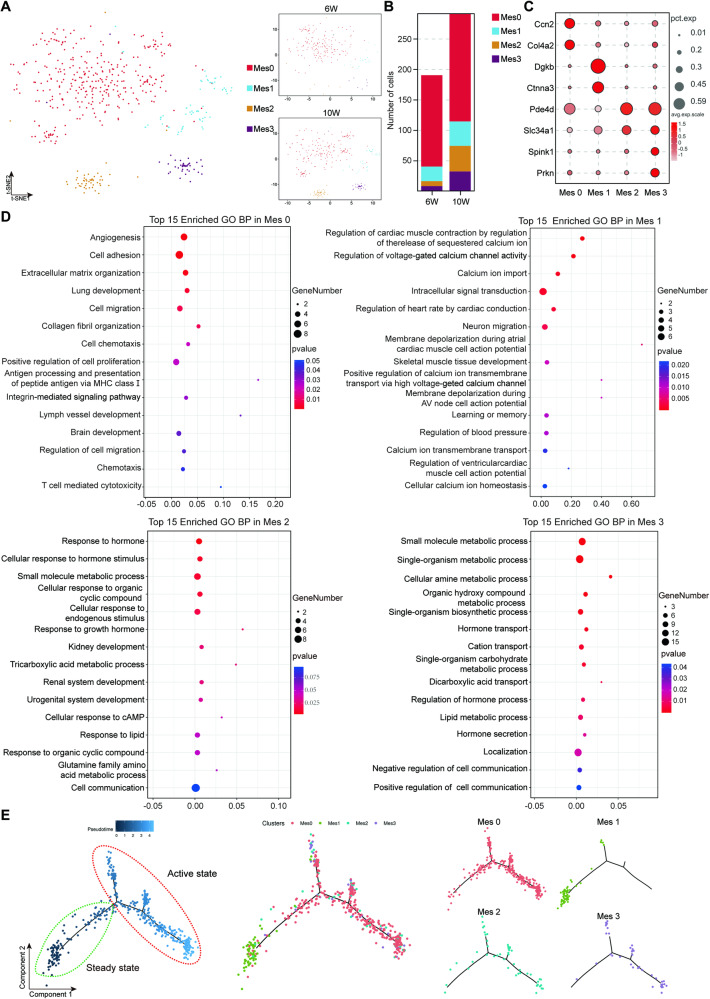
Molecular and temporal profiles of MC subtype heterogeneity. **A** t-SNE analysis showing that there were four subclusters of MCs. Different cell subclusters are color-coded. **B** Changes in the numbers of cells in each mesangial subcluster in both the 6- and 10-week samples. **C** Bubble plots showing the expression of marker genes in subclusters of MCs. **D** Top 15 enriched GO BP terms of DEGs in Mes 0–3. **E** Distribution of MCs in the cell trajectory: the right panel is the pseudo-time trajectory of each MC sub-cluster.

MCs, important intrinsic cells of the glomerulus, play an important role in the maintenance of renal function. Therefore, we performed an unbiased cluster analysis of 481 MCs and identified four subclusters of MCs (Mes 0–3) (Fig. 3A). Mes 0 was the dominant cell population, accounting for more than 60% of the entire MC population (Fig. 3B). From 6 to 10 weeks, the numbers of Mes 0–3 all increased, with more significant increases in Mes 2 and Mes 3 clusters. Marker gene bubble maps were constructed for the four subclusters of MCs (Fig. 3C). In addition, the Gene Ontology (GO) enrichment of the biological process (BP) category among the upregulated genes of the MC clusters is shown in Fig. 3D. Mes 0 was mainly involved in cell adhesion and collagen fiber production. Mes 1 was mainly involved in calcium ion transport and muscle contraction. Mes 2–3 were mainly involved in response to hormones, metabolic processes, and cellular communication, which were related to the maintenance of glomerular homeostasis. Next, we carried out the pseudo-time analysis of MCs. As shown in Fig. 3E, the MCs were divided into two stages, steady state and active state. Mes 1 was almost in the steady state, while Mes 0, Mes 2, and Mes 3 were almost in the active state. These results coincided with the GO BP analysis described above. Renal cell heterogeneity in C57BL/6 mice as well as MCs subclusters were shown in Supplementary Fig. 1.

### Macrophages communicate with MCs via the CXCL12/DPP4 axis

**Fig. 4:**
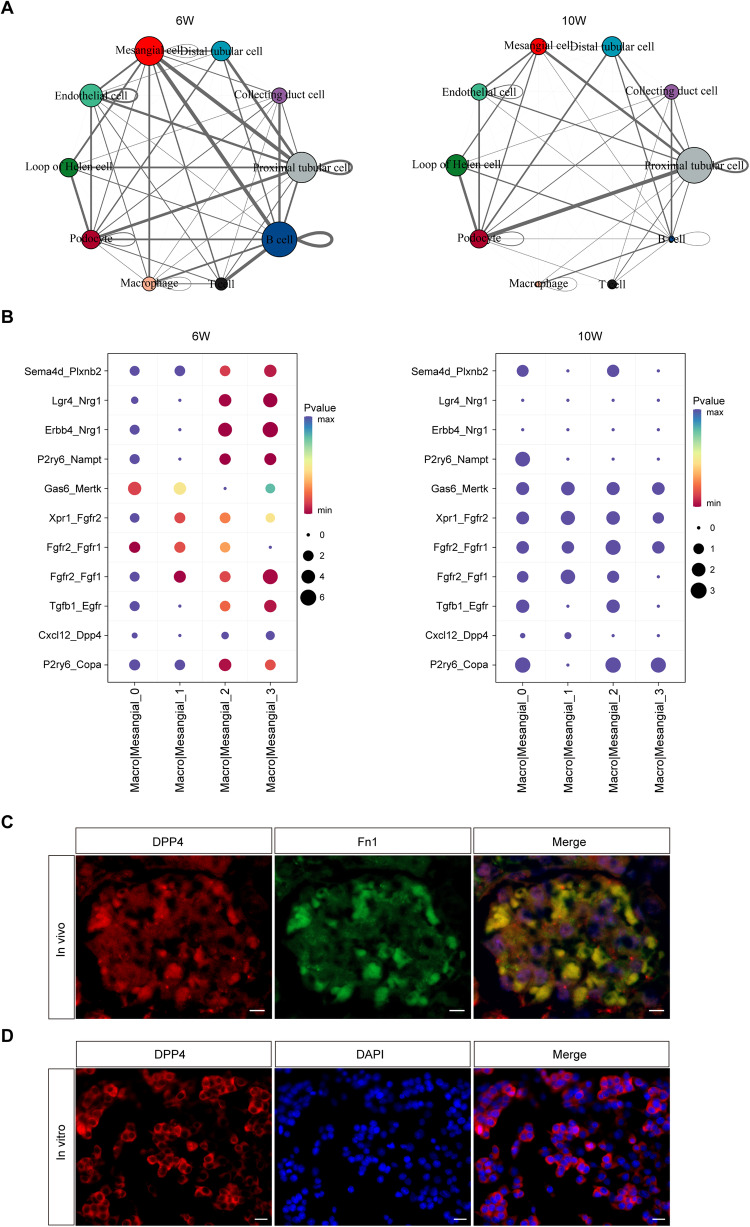
Cell–cell communication networks in the kidneys of LN mice by CellPhoneDB analysis. **A** Network visualization of ligand–receptor connectivity in LN mice. Nodes represent clusters; the larger the node, the more the interactions between the cell and other cell types. The lines represent the interactions between nodes, and the thickness of each line is proportional to the strength of the ligand–receptor pair between cell types. **B** Ligand–receptor relationship between macrophages and MC subclusters when MCs are the receptor cells. **C** Immunohistochemical co-localization of Fn1 and DPP4 in the kidneys of LN mice. Scale bar = 5 μm, *n* = 5 for each group. **D** DPP4 immunofluorescence staining of DPP4 in glomerular MCs. Scale bar = 20 μm, *n* = 5 for each group.

In the course of LN, the deposition of IC activates intrinsic cells, such as MCs and endothelial cells and activates macrophages toward sites of injury through the secretion of various factors, including IL-6 and transforming growth factor-β (TGF-β). At the same time, nitric oxide (NO) secreted by macrophages further stimulates the activation of renal intrinsic cells. To evaluate these effects, a cell-cell communication network was constructed from 6- and 10-week samples. The nodes represented different cell types, and the edges indicated the interactions between cell types. As shown in Fig. 4A, there was a strong reciprocal relationship between macrophages and MCs at 6 weeks, though the interaction was weakened at 10 weeks. Figure 4B further demonstrated the ligand-receptor interactions between macrophages and subclusters of MCs when MCs functioned as receptors. Current research has shown that the CXC chemokine family plays important roles in LN. CXCL13 promoted the proliferation of human renal MCs, and CXCL10 overexpression correlated with clinical LN and showed utility as a new marker to assess renal disease activity in Chinese patients with pediatric SLE [32, 33]. Therefore, we focused on the CXCL12/DPP4 signaling axis. Macrophages secreted CXCL12 to regulate the DPP4 ligand on Mes 2-3 at 6 weeks, while this relationship was significantly weakened at 10 weeks. Hence, we speculated that this ligand–receptor played an important role in the early stage of LN. We then performed co-localization staining of Fn1 (a classical marker for MCs) and DPP4 in the kidneys of LN mice (Fig. 4C) and verified the expression of DPP4 in the MC cell line SV40-MES-13 (Fig. 4D). The results revealed that DPP4 was a membrane protein expressed in MCs. Cell–cell communication in the kidney of C57BL/6 mice is shown in Supplementary Fig. 2.

### CXCL12 promotes the proliferation and migration of MCs via DPP4 in vitro

**Fig. 5:**
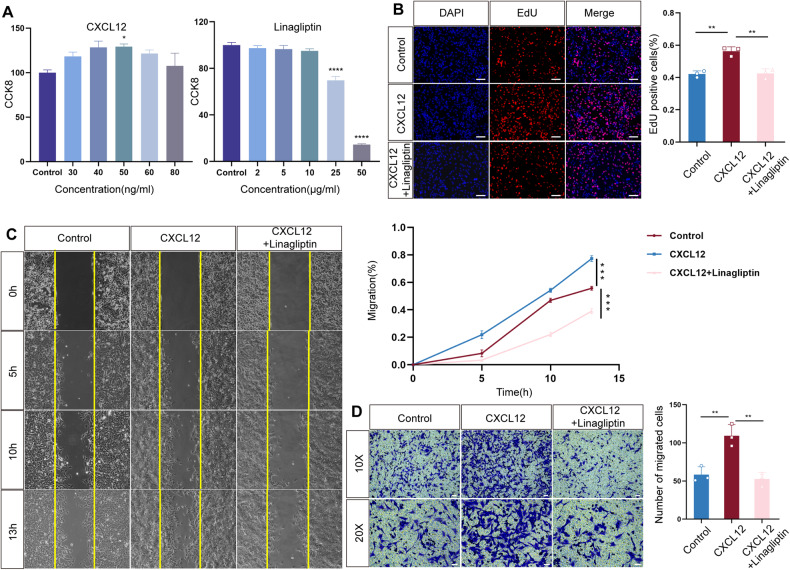
Macrophages regulate MCs via the CXCL12/DPP4 axis. **A** CCK8 assays showing the effects of CXCL12 and linagliptin on MC viability. **B** EdU assays were performed to detect the proliferation of MCs treated with CXCL12 and linagliptin. Scale bar = 75 μm, *n* = 3 for each group. **C** Wound healing and **D** Transwell assays were performed to detect the migration of MCs treated with CXCL12 and linagliptin. 10×: Scale bar = 75 μm. 20×: Scale bar = 50 μm, *n* = 3 for each group.

MC hyperproliferation is one of the representative pathological changes in the progression of LN. MCs can also migrate to endothelial cells to destroy the glomerular structure [30]. Therefore, we detected the proliferation and migration of MCs after treatments with CXCL12 and a DPP4 inhibitor in vitro. DPP4 inhibitors block the breakdown of glucagon-like peptide-1 (GLP-1) and glucose-dependent insulinotropic polypeptide (GIP) to increase the levels of active hormones (hereafter, all are referred to as linagliptin) [34]. Linagliptin is a class drug for the treatment of type 2 diabetes mellitus that can promote the release of insulin from pancreatic islet β-cells and inhibit the secretion of glucagon by pancreatic α-cells. After Cell Counting Kit 8 (CCK8) testing was performed to verify cell viability, as shown in Fig. 5A, we chose concentrations of 50 ng/mL CXCL12 and 10 μg/mL linagliptin for further analyses. To detect their effects on MC proliferation capacity, we performed a 5-ethynyl-2′-deoxyuridine (EdU) assay. The percentage of EdU-labeled proliferating cells increased significantly after adding CXCL12, while linagliptin was able to restore the proliferation induced by CXCL12 (Fig. 5B). Meanwhile, we explored the effect of the CXCL12/DPP4 axis on MC migration in wound healing and Transwell assays. The data showed that CXCL12 can promote MC migration compared with that of the control group. In contrast, the migration ability was notably attenuated after adding linagliptin to the culture medium (Fig. 5C, D). The above results indicated that CXCL12 could regulate the proliferation and migration abilities of glomerular MCs through DPP4.

### Interference of the CXCL12/DPP4 axis inhibits MC proliferation and migration

**Fig. 6:**
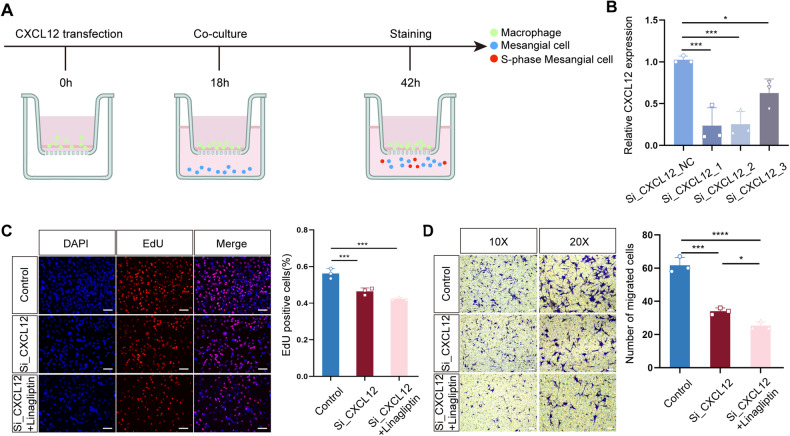
CXCL12 promotes the proliferation and migration of MCs via DPP4 in vitro. **A** Co-culture pattern map of macrophages and MCs. **B** qRT-PCR assays were performed to detect the expression of CXCL12 in macrophages after siRNA interference. **C** EdU assays were performed to characterize the proliferation of MCs when knocking down CXCL12 in macrophages and adding linagliptin. Scale bar = 75 μm, *n* = 3 for each group. **D** Transwell assays were performed to characterize the migration of MCs when knocking down CXCL12 in macrophages and adding linagliptin. 10×: Scale bar = 75 μm. 20×: Scale bar = 50 μm, *n* = 3 for each group.

To further validate the regulation of macrophages on MCs, we co-cultured MCs and macrophages for 18 h in cell chambers (Fig. 6A). We first knocked down CXCL12 in the RAW 264.7 macrophage cell line with small interfering RNA (siRNA). The results showed that the expression of CXCL12 in macrophages was effectively suppressed by all three CXCL12 siRNAs (Fig. 6B). We then performed EdU and Transwell assays. Interfering with CXCL12 in macrophages inhibited the proliferation of MCs (Fig. 6C). The interference, combined with linagliptin treatment, further reduced MC proliferation. The Transwell assay results showed that knockdown of CXCL12 in macrophages significantly inhibited MC migration, and the addition of linagliptin aggravated this phenomenon (Fig. 6D). These data suggested that macrophages can regulate glomerular MC proliferation and migration through the CXCL12/DPP4 axis.

### Linagliptin decelerates the progression of LN mice

**Fig. 7:**
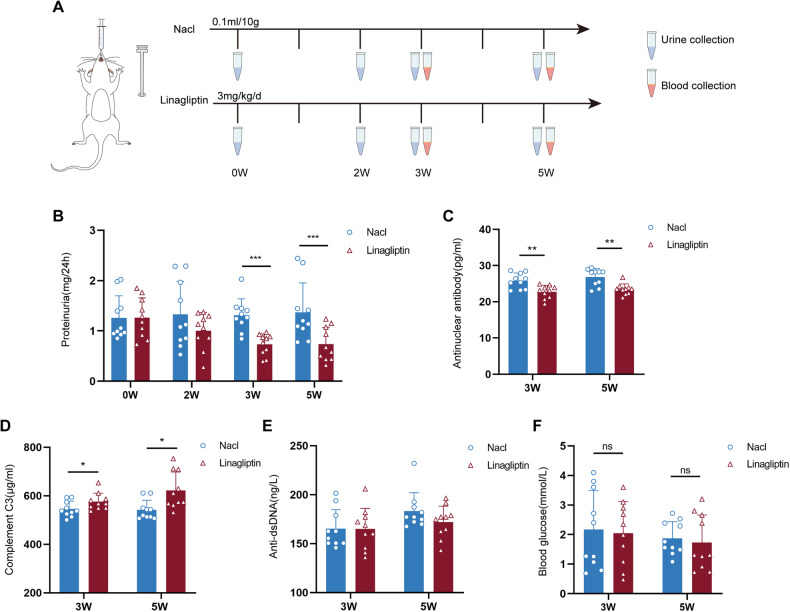
Therapeutic effect of linagliptin on LN mice. **A** Flowchart of LN mice treated with saline and linagliptin. Linagliptin treatment group (*n* = 10 for each group) (3 mg/kg/d) and control group (*n* = 10 for each group) (0.1 mL/10 g). **B** Quantitative analysis of 24-h proteinuria in LN mice after 0, 2, 3, and 5 weeks of gavage treatment. **C**–**E** Serum levels of ANA, complement C3 and anti-dsDNA in the treatment and control groups after 3 and 5 weeks of gavage treatment. **F** Blood glucose levels in the treatment and control groups.

**Fig. 8:**
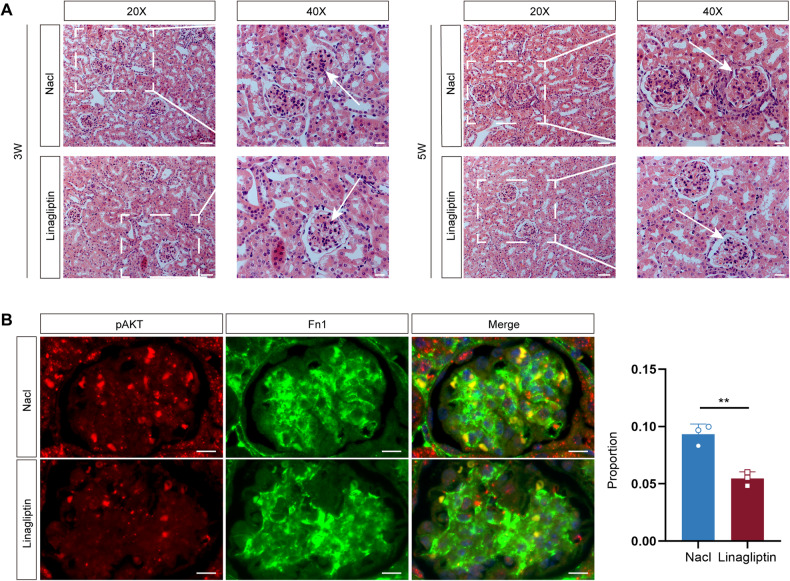
HE staining and pAKT expression in the kidney of LN mice after gavage treatment. **A** HE staining of kidneys in the treatment and control groups after 3 and 5 weeks of gavage treatment. 20×: Scale bar = 50 μm. 40×: Scale bar = 20 μm, *n* = 10 for each group. **B** The expression of pAKT on MCs was verified by co-immunolocalization staining with Fn1. Scale bar = 10 μm, *n* = 3 for each group.

**Fig. 9:**
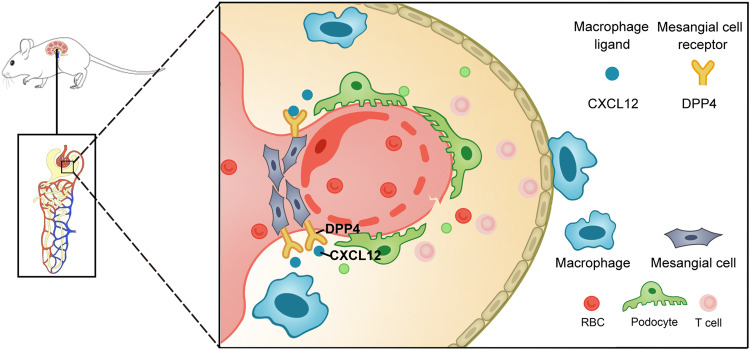
Schematic illustrating the interaction between macrophages and glomerular MCs in LN. In the early stage of LN, macrophages regulate MCs through the CXCL12/DPP4 axis to initiate the pathogenesis and affect MC proliferation and migration. As the disease progresses, the proliferation and migration of the MCs and endothelial cells will damage the structure and function of the glomerulus, block Baumann’s capsule, affect the glomerular filtration function, destabilize the microvascular endothelial stability, aggravate hematuria, and ultimately lead to renal injury.

To reveal the therapeutic effect of linagliptin on LN, we treated LN mice with linagliptin (3 mg/kg/d) and compared the effects with a control group that was gavaged with saline (0.1 mL/10 g) (Fig. 7A). We collected urine samples from the mice at 0, 2, 3, and 5 weeks after linagliptin treatment and tested the 24-h urinary protein levels. After 3 weeks, the quantitative urinary protein levels were 0.7317 ± 0.2092 mg/24 h in the linagliptin-treated group and 1.314 ± 0.3215 mg/24 h in the control group. After 5 weeks, the quantitative urinary protein levels were 0.7369 ± 0.3344 mg/24 h in the linagliptin-treated group and 1.327 ± 0.6131 mg/24 h in the control group (Fig. 7B), indicating that linagliptin treatment markedly reduced the level of proteinuria. Further, we collected blood serum samples at 3 and 5 weeks after linagliptin treatment and performed enzyme-linked immunosorbent assays (ELISAs) to detect the levels of ANA, anti-dsDNA antibody, and complement C3. Compared with the control group, after 3 weeks, the level of ANA was 22.74 ± 1.781 pg/mL, that of complement C3 was 576.2 ± 34.57 μg/mL, and that of anti-dsDNA was 164.9 ± 21.11 ng/L in the linagliptin group (the level of ANA was 25.79 ± 2.146 pg/mL, that of complement C3 was 545.8 ± 32.00 μg/mL, and that of anti-dsDNA was 165.3 ± 19.56 ng/L in the control group). After 5 weeks, the level of ANA was 23.45 ± 1.490 pg/mL, that of complement C3 was 622.5 ± 76.03 μg/mL, and that of anti-dsDNA was 172 ± 16.33 ng/L in the linagliptin group (the level of ANA was 26.82 ± 2.358 pg/mL, that of complement C3 was 541.9 ± 39.77 μg/mL, and that of anti-dsDNA was 183.3 ± 18.78 ng/L in the control group). These results showed that linagliptin reduced ANA and anti-dsDNA antibody concentrations and increased complement C3 levels in LN mice (Fig. 7C–E). The HE staining results revealed that glomerular MC proliferation, the increase in extracellular matrix, inflammatory cell infiltration, and tubular dilatation were attenuated in the linagliptin-treated group compared with the control group (Fig. 8A). It has been found that DPP4 inhibitors exert their effects are not directly, and the use of DPP4 inhibitors does not affect the expression of DPP4 on tissues [35, 36]. Our experiment also demonstrated that compared with the control group, linagliptin treatment did not affect the expression of DPP4 on glomeruli, which was shown in Supplementary Fig. 3. In addition, AKT signaling pathway is closely related to cell proliferation, and studies have found that inhibition of AKT/mTOR signaling pathway inhibited mesangial cell proliferation and fibrosis [37, 38]. Therefore, we examined the activation of pAKT in mesangial cells in the control and treated groups. As shown in Fig. 9B, we performed immunohistochemical co-localization staining for the mesangial cell marker gene Fn1 and pAKT. The results showed that pAKT expression in mesangial cells was decreased in the treatment group compared with the control group. This suggested that DPP4 inhibitors could inhibit mesangial cell proliferation by down-regulating the AKT signaling pathway. Linagliptin is commonly used clinically in the treatment of type 2 diabetes mellitus in an oral form, and it has been shown that the risk of hypoglycemia caused by the DPP4 inhibitor is very low [39]. Our experiments also showed that the blood glucose levels in the treatment group were reduced compared with the control group; however, there was no statistically significant difference between them (Fig. 7F). These results indicated that linagliptin would not cause the side effect of hypoglycemia.

## Discussion

SnRNA-seq has been widely used to characterize the heterogeneity of various cell types in different organs and to study the pathological processes of diseases. However, there have been no reports of the use of snRNA-seq in elucidating the relationship between MCs and inflammatory cells. Here, we used this technology to identify the heterogeneity and subclusters of MCs in the kidneys of LN mice. We then focused on the molecular interactions between macrophages and MCs and found that macrophages regulated the proliferation and migration of glomerular MCs through the CXCL12/DPP4 axis in LN.

With the development of single-cell RNA sequencing (scRNA-seq), an increasing number of studies have used this technology to identify renal issues in developmental or pathological states. Arnon Arazi et al. identified 21 disease-active leukocyte subpopulations and two chemokine receptors, CXCR4 and CX3CR1, that were critical for the development of LN using scRNA-seq [40]. Chung et al. found that CCL2 was consistently highly expressed and that 80% of CCL2 came from damaged MCs in nephrotoxic serum-induced LN mice, indicating that damaged MCs are an important driver of inflammation [41]. Zhou et al. described the possibility of using mesenchymal stem cells (MSCs) to treat LN. By scRNA-seq, MSCs were found to reduce pro-inflammatory T cells and macrophages in the renal region of LN, meanwhile, anti-inflammatory T cells were increased [42]. However, we used snRNA-seq instead of scRNA-seq for our analysis. Although snRNA-seq and scRNA-seq have comparable genetic detection sensitivity, research has shown that snRNA-seq results from sNuc-DropSeq, DroNc-seq, and 10X Chromium platforms can capture a diversity of kidney cell types that are not represented in the scRNA-seq dataset, including glomerular podocytes, MCs, and endothelial cells. Several other advantages are offered by snRNA-seq, including reduced dissociation bias, the elimination of dissociation-induced transcriptional stress responses, and the ability to successfully detect inflamed fibrotic kidneys [43–45].

MCs originate from forkhead box D1 (Foxd1) precursor cells and account for nearly one-third of the total number of glomerular cells [46, 47]. In addition to secreting growth factors, MCs can also generate cellular matrix with microfibers that anchor to the basement membrane, thereby controlling capillary pressure and stability to produce suitable glomerular filtration and plasma ultrafiltration rates [48]. Moreover, studies have found that MCs can participate in the immune response by secreting inflammatory mediators [49, 50]. In general, when IC are deposited in MCs, the cells will suffer persistent and severe damage. On the one hand, MCs continuously proliferate and produce a large amount of extracellular matrix [30]. On the other hand, MCs under pathological conditions secrete C–C motif chemokine ligand 2 (CCL2), IL-6, and IFN, which stimulate the activation of macrophages, their transformation to the M1 type, and the secretion of TNF-α. Notably, activated immune cells migrate to the site of injury and generate IL-6, interferon-induced protein 35 (IFI35), hypoxia-inducible factor-1α (HIF-1α), CXCL10, CXCL13, and glycosaminoglycans, which can cause secondary damage to MCs [51, 52]. In addition to inflammatory factors, several endogenous inflammatory signaling pathways (e.g., nuclear factor-κB (NF-κB), mitogen-activated protein kinase (MAPK), c-Jun terminal kinase (JNK), and protein kinase B (AKT)) have also been identified, which induce the hyperactivation of renal MCs and immune cells [30, 53]. All the above indicate that glomerular MCs have strong plasticity and play an important role in the occurrence and development of renal diseases. Once the MCs are injured, they can directly or indirectly affect renal function, ultimately leading to renal failure [46]. Therefore, targeting MCs or blocking the interaction between inflammatory cells and MCs may become a good therapeutic approach for LN.

Our research revealed that macrophages could regulate DPP4 receptors on MCs by secreting CXCL12. DPP4 inhibitors are a class of hypoglycemic drugs used in type 2 diabetes. Some studies have shown that DPP4 inhibitors can alleviate kidney diseases, such as diabetic nephropathy, hypertensive nephropathy, and crescentic nephritis [35, 54, 55]. However, there have been no studies using DPP4 inhibitors in the treatment of LN. In our study, we treated LN mice with linagliptin. The results showed that mice in the linagliptin-treated group exhibited significantly less renal inflammation compared to the control group. Moreover, linagliptin treatment significantly reduced the levels of urinary protein, ANA, and anti-dsDNA antibody and elevated complement C3 levels in LN mice. Therefore, linagliptin can attenuate renal injury and delay the disease progression of LN. It is noteworthy that linagliptin metabolized through the intestine does not aggravate the renal burden or cause side effects such as hypoglycemia. Our study demonstrated the potential feasibility of linagliptin for the treatment of LN and provided a new avenue for the clinical treatment of LN.

The efficacy of linagliptin as a monotherapy in the treatment of LN has been successfully validated. In future studies, our goal is to explore combinations of glucocorticoids with linagliptin and/or immunosuppressants for treating LN. The implementation of these measures will contribute to enhancing the effectiveness of linagliptin while reducing the reliance on glucocorticoids and minimizing side effects, such as infections, osteoporosis, and obesity.

In conclusion, we defined the heterogeneity of kidney cells in LN mice and the interaction between macrophages and MCs by snRNA-seq. We revealed that macrophages communicated with MCs through the CXCL12/DPP4 axis, and this ligand–receptor interaction played an important role in the development of LN. Our research confirmed that interfering with CXCL12 or treating LN mice with linagliptin could inhibit MC proliferation and migration and slow down the development of LN, providing a new target and solution for the treatment of LN.

## Materials and methods

### Animals

Female MRL/lpr mice at 4, 6, 10, 12 weeks were provided by Shanghai SLAC Laboratory Animal Company. All animals were maintained and used in accordance with the guidelines of Institutional Animal Care of Nantong University. All animal experiments were approved by the Institutional Animal Ethics Committee of Nantong University (approval number: S20231209-001). Animals were housed in temperature-controlled (24 °C) and humidity-controlled (50%) animal quarters with a 12 h light–dark cycle and had free access to food and water.

### Isolation of renal cortex and preparation of cell nucleus suspension

At 6 and 10 weeks after LN induction, mice were anesthetized and perfused. Each group has 5 mice. The renal cortexes were collected, minced, and lysed. One milliliter of filtered tissue homogenate was then mixed with 1 mL 50% iodixanol (DKSC) (D1556-250ML, MERCK, Darmstadt, Germany). The tissue mixture was added slowly into 3 mL of 33% DKSC in a 15-mL centrifuge tube for gradient centrifugation. After centrifuging at 3234 × *g* for 20 min at 4 °C, 3.5 mL of the upper layer of the centrifuged solution was transferred to a new 15-mL centrifuge tube. Then, the 1-mL cell nucleus layer at the interface of the gradient centrifugation was transferred to a new 15-mL centrifuge tube, and 2 mL of nuclear cleaning solution was added to resuspend the buffer. Finally, we used a microscope cytometry plate stain with 0.4% trypan blue to detect the concentration, viability, and integrity of the nuclei.

### Construction and sequencing of the scRNA-seq library

Nuclear suspensions from five mice in each group were combined and loaded on the 10X Genomics GemCode Single-cell instrument and processed following the manufacturer’s protocol to produce single-cell Gel Bead-In-EMulsions (GEMs). According to the manufacturer’s instructions, we then constructed the sequencing libraries with the Chromium Next GEM Single Cell 3′ Reagent Kit v3.1 (10X Genomics company), which contained a read sequencing primer, a 10X barcode, a unique molecular identifier (UMI), and poly-dT primer. Cell lysis, RNA extraction, cDNA synthesis, and amplification were automatically completed on the Illumina sequencing platform (Genedenovo Biotechnology, Guangzhou, China).

### Alignment of snRNA-seq reads

The raw BCL files were converted to FASTQ files, and then individual samples were aligned and quantified using 10X Genomics Cell Ranger software (version 3.1.0). Briefly, reads with low-quality barcodes and UMIs were filtered out and then mapped to the reference genome. Quality control (QC) filters were applied using the following parameters, similar to what has been reported [45, 56]. First, at least 50% of reads mapping to the transcriptome and intersecting an exon were considered. Second, cells with unusually high numbers of UMIs (≥5000) or mitochondrial gene percentages (≥25%) were filtered out. Third, we excluded cells with less than 200 or more than 8000 genes detected. After the QC filters, a total of 39,074 cells from independent experiments were analyzed by Seurat software.

### Dimension reduction and cell clustering

To minimize the influences of batch effect and behavioral conditions on clustering, Seurat, which utilizes canonical correlation analysis and mutual nearest neighbor analysis, was used to aggregate all samples [57]. Principal component analysis (PCA) dimension reduction was performed on the merged data [58]. Cells were clustered using graph-based clustering of the PCA-reduced data with the Louvain method after computing a shared nearest neighbor graph [59]. For sub-clustering, we applied the same procedures of scaling, dimensionality reduction, and clustering to specific data sets (such as MCs). For the visualization of clusters and sub-clusters, t-SNE plots were generated [60].

### Identification of DEGs and GO enrichment analysis

Differential gene expression analysis of MCs from normal control and LN mice was performed using the Wilcoxon rank-sum test. Based on the subgroup information and sample information to which the cells belonged, Seurat software was used to analyze the differences between groups of cells. Briefly, DEGs were identified using several criteria: 1) genes had to be at least 1.28-fold overexpressed in the target cluster, 2) genes had to be expressed in more than 25% of the cells in the target cluster, and 3) *P* value < 0.01 [61].

### GO enrichment analysis

GO enrichment analyses for the DEGs were then performed using the Gene Ontology database (www.geneontology.Org), which provides all GO terms that are significantly enriched for DEGs compared to the genomic background, as well as filters DEGs corresponding to their biological functions [62].

### Construction of single-cell trajectories

The single-cell trajectories on sub-clustered MCs and MCs of four samples were analyzed by Monocle (Version 2.6.4) [63]. Monocle reduces the space to 2D and sorts the cells. Once the cells were sorted, we were able to visualize the trajectories in a reduced-dimensionality space. The trajectories had a tree-like structure, including tips and branches.

### Prediction of cellular crosstalk

Differential ligand–receptor assays were performed separately for cells from normal and LN mice. We analyzed the expression abundance of ligand–receptor interactions between macrophages and MCs by CellphoneDB, currently the most widely used cell communication analysis software available [64]. Among them, only receptors and ligands that were expressed at levels greater than 10% were considered for the analysis [65].

### Immunofluorescence multiple staining for co-localization studies

Tissue sections were incubated with anti-Fhl2 antibody (1:400, ab202584, Abcam, Cambridge, UK) and anti-Lama2 antibody (1:400, ab11576, Abcam, Cambridge, UK) at 4 °C overnight. After incubation with secondary antibodies for 2 h, the sections were sealed with antifade mounting medium with 4′,6-diamidino-2-phenylindole (DAPI, 041221210930, Beyotime, Shanghai, China). Images were acquired by a Zeiss AX10 microscope (Carl Zeiss, Weimar, Germany).

### Hematoxylin and eosin (HE) staining and immunohistochemical analysis

Kidney tissues were collected and fixed in 4% paraformaldehyde for 24 h. After gradient dehydration, the kidney tissues were embedded in paraffin and finally cut into 3-μm sections. The HE staining was performed with a Hematoxylin and Eosin Staining Kit (Beyotime, Shanghai, China). Referring to the evaluation criteria from previous literature, we scored the pathological conditions of the glomerulus and interstitial cells by selecting different visual fields. Morphometric analyses of the glomerulus and renal tubules were determined using ImageJ. For immunostaining, the sections were incubated with anti-Fhl2 antibody (ab202584, Abcam, Cambridge, UK), anti-Lama2 antibody (ab11576, Abcam, Cambridge, UK), anti-DPP4 antibody (GB114937, Servicebio, Wuhan, China), anti-Fn1 antibody (GB114491, Servicebio, Wuhan, China) and pAKT (4060S, Cell Signaling Technology, Boston, MA, USA). After incubation with secondary antibodies for 2 h, the sections were sealed with antifade mounting medium with DAPI (041221210930, Beyotime, Shanghai, China). Images were acquired by a Zeiss AX10 microscope (Carl Zeiss, Weimar, Germany).

### Cell culture

The MC line SV40-MES-13 and the macrophage cell line RAW 264.7 (ATCC, Beijing, China) were respectively cultured in Dulbecco’s Modified Eagle Medium F12 (DMEM-F12, Gibco, Grand Island, NY, USA) and DMEM (Gibco, Grand Island, NY, USA) supplemented with 10% fetal bovine serum (FBS) (Gibco, Grand Island, NY, USA) and 1% penicillin–streptomycin (Life Technologies, Carlsbad, CA, USA) under 5% CO_2_ at 37 °C.

### CCK8 assay

Cells were seeded in 96-well plates, and 100 μL of medium was added to each well. After complete attachment, CXCL12 (Minneapolis, MN, USA) and linagliptin (Boehringer Ingelheim, Shanghai, China) were added, and the cells were cultured for 24 h. Cells were incubated in a 100-μL reaction mixture (10 μL CCK-8 and 90 μL DMEM–F12 or DMEM) (CCK-8, Beyotime, Shanghai, China) for 2 h and then 96-well plates was measured at a wavelength of 450 nm.

### EdU incorporation assay

Cells (6000/well) were adhered to 96-well plates. After 24 h of incubation, 1 μM EdU (Ribobio, Guangzhou, China) was added to each well and incubated for 2 h at 37 °C. Cells were then permeabilized with 0.5% Triton X-100 for 10 min after being fixed with 4% paraformaldehyde for 30 min at 37 °C, followed by EdU staining with the Apollo staining reaction for 30 min. Subsequently, Hoechst stain was added to visualize the nuclei for 30 min, and the proportion of nucleated cells stained with EdU was observed under a fluorescence microscope.

### Wound healing assay

Cells were seeded in chambers, which were pulled out vertically after all the cells had adhered to the walls and grew to confluency. The chambers were then rinsed with phosphate-buffered saline (PBS) two times, and complete medium containing the appropriate drug was added. Images of the cells were then captured with a microscope at 0, 5, 10, and 13 h. The wound closure was calculated according to the following formula: (initial wound size-current wound size)/ initial wound size× 100.

### Transwell assay

Cells (3000/well) were seeded in upper chambers with 100 μL of medium without FBS. Then, Transwell chambers (Corning, NY, USA) were placed in 24-well plates containing medium, with 500 μL of medium with 10% FBS in the bottom chambers. After incubation at 37 °C for 24 h, the cells on the upper sides of the chambers had migrated to the lower surfaces of the chambers, and the chambers were fixed with 4% paraformaldehyde and stained with 0.1% crystal violet solution. Images were captured using a microscope.

### siRNA knockdown of CXCL12

CXCL12 was knocked down by siRNA (Ribobio, Guangzhou, China), which are listed in Supplementary Table 1. The siRNAs were ordered from Rebio and transfected into RAW 264.7 cells.

### RNA extraction and real-time reverse-transcription (RT) PCR

Total RNA was extracted by using TRIZOL reagent (Life Technologies, Carlsbad, CA, USA). The total RNA was then reverse transcribed to obtain cDNA using an RT-PCR kit (Promega, Madison, WISC, USA). Then, the cDNA was amplified with specific primers using an SYBR Green PCR Kit (Qiagen, Dusseldorf, Germany). Primer sequences are provided in the Supplementary table 2.

### In vivo intragastric administration assay

Forty LN mice were randomly divided into four groups, 10 in each group, and treated with normal saline (0.1 mL in 10 g) and linagliptin (Boehringer Ingelheim, Shanghai, China) (3 mg/kg/d). Two groups were treated for 3 weeks, and the remaining groups were treated for 5 weeks. The 24-h urine samples were collected at 0, 2, 3, and 5 weeks, and the serum and kidney tissues were collected at 3 and 5 weeks. No mice were excluded from the analysis.

### Twenty-four-hour urine protein measurement

For the collection of urine samples, the mice were placed in a metabolic cage 1 day in advance. LN 24-h urine samples were collected 1 day later. Urine was collected in 10-mL Eppendorf tubes and stored at −20 °C. After the samples were collected at each time point, the urine proteins were measured by an automatic biochemical detector (Au5800, Beckman Coulter, CA, USA).

### ELISA

After 3 and 5 weeks of intragastric administration, serum, kidneys, and other tissues were collected from LN mice. After the mice were anesthetized, excess fur was cut off, and blood was taken from behind the eyeball. The blood samples were then centrifuged at 1000 rpm, and the supernatant sera were obtained for ELISA. The concentrations of anti-dsDNA IgG, ANA IgG, and complement C3 in serum samples were measured using ELISA kits (JM-11451M2, JINGMEI, Yancheng, China; JM-03001M2, JINGMEI, Yancheng, China; JM-02637M1, Yancheng, JINGMEI, China) according to the manufacturer’s instructions.

### Blood glucose testing

Blood samples were centrifuged at 1000 rpm, and the supernatants were placed in an automatic biochemical detector (Au5800, Beckman Coulter, CA, USA) to measure blood glucose values.

### Statistical analysis

All experiments were repeated at least three times. SPSS v24.0 (IBM, SPSS, Chicago, IL, USA) and Graphpad Prism 9 (GraphPad Software, La Jolla, CA, USA) were used to perform statistical analyses. Normally distributed data presented as mean ± standard deviation (SD), and non-normally distributed data represented by median and interquartile range. Differences between the two groups were analyzed by Student’s *t*-test. *P*-value of <0.05 was considered significant: *p < 0.05, **p < 0.01, and ***p < 0.001.

### Supplementary information

Supplemental materials

## Data Availability

The data and material are available by contacting the corresponding author upon reasonable request.
