# Supplementary material for: Macrophages communicate with mesangial cells through the CXCL12/DPP4 axis in lupus nephritis pathogenesis
Source: Cell Death Dis. 2024 May 18;15(5):344. doi: 10.1038/s41419-024-06708-4 (PMC11102518; doi:10.1038/s41419-024-06708-4)
Supplement: Supplementary file 1 — Supplemental materials [file 41419_2024_6708_MOESM1_ESM.docx]

**Supplementary figures：**


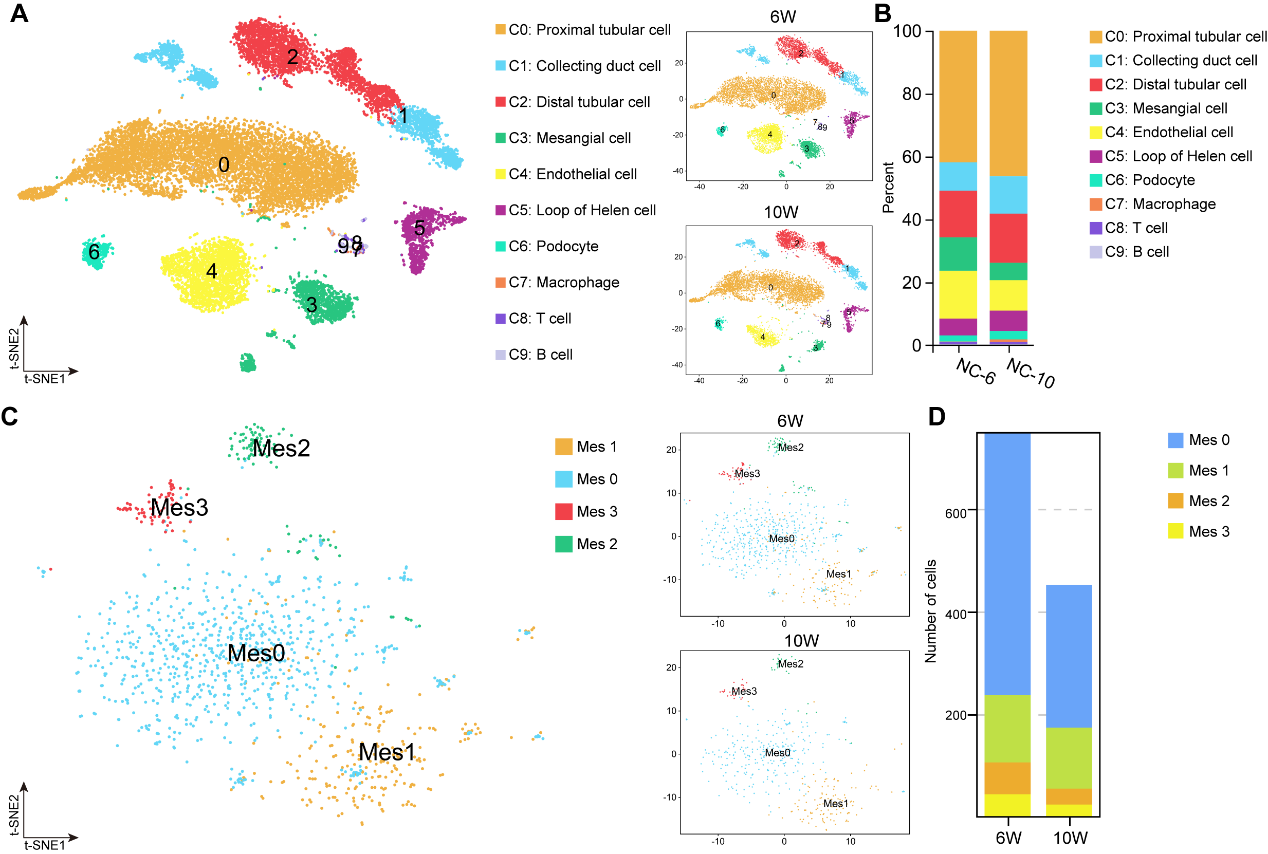


**Figure S1. SnRNA-seq analysis revealed cellular heterogeneity in the kidneys of C57BL/6 mice.**

(A) t-SNE analysis (left) showed that there were 10 different cell clusters in the renal cortex. The distributions of renal cells at 6 and 10 weeks (right). Different cell clusters are color-coded. (B) Changes in the proportions of each cell cluster from 6 to 10 weeks. (C) t-SNE analysis showing that there were four subclusters of MCs. Different cell subclusters are color-coded. (D) Changes in the numbers of cells in each mesangial subcluster at both 6 and 10 weeks.


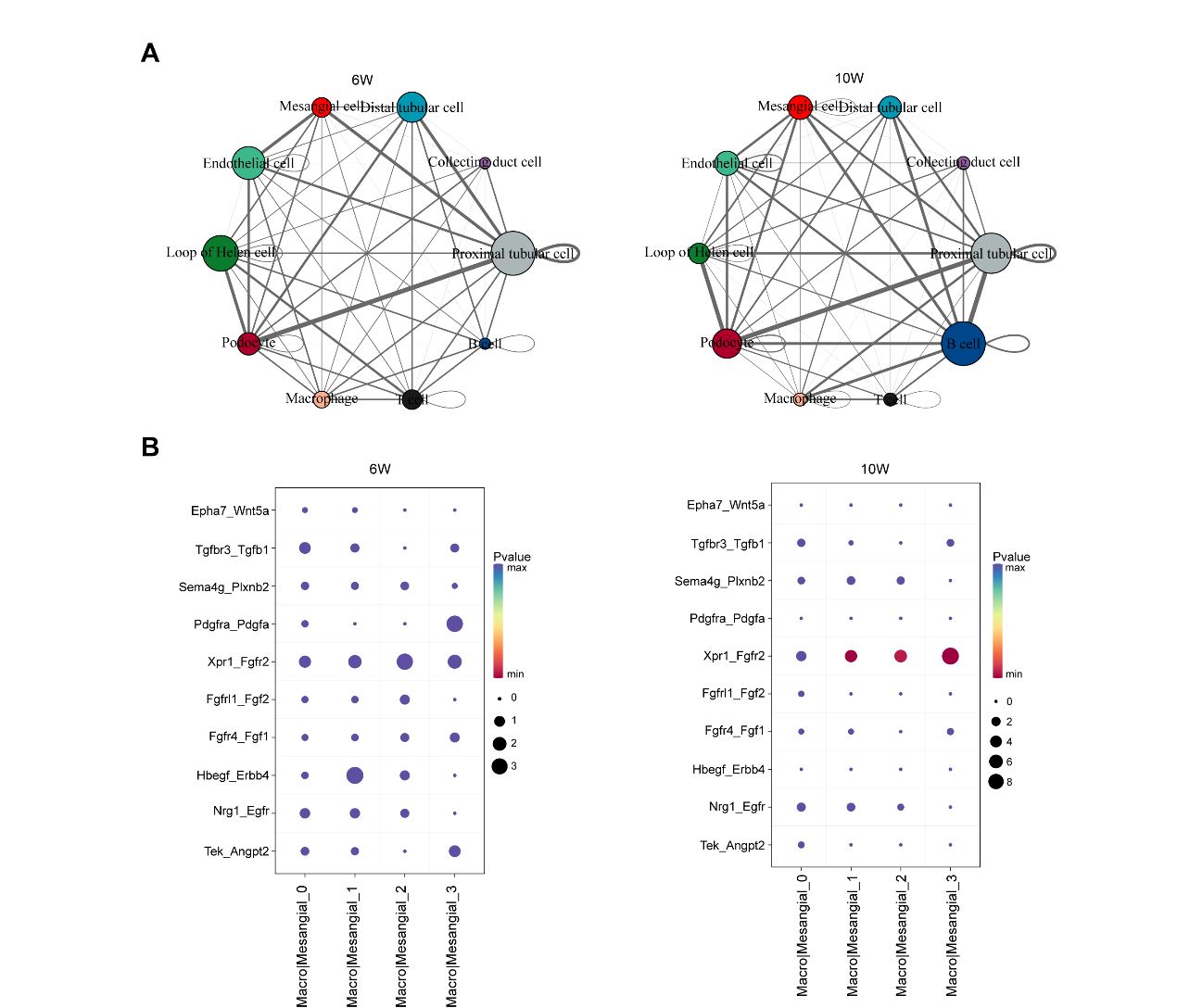


**Figure S2. Cell-cell communication networks in the kidneys of C57BL/6 mice by CellPhoneDB analysis.**

(A) Network visualization of the ligand–receptor connectivity in C57BL/6 mice. Nodes represent clusters; the larger the node, the more interactions between the cell and other cell types. The lines represent the interactions between nodes, and the thickness of each line is proportional to the strength of the ligand–receptor pair between cell types. (B) Ligand-receptor relationship between macrophages and MC sub-clusters when MCs are the receptor cells.


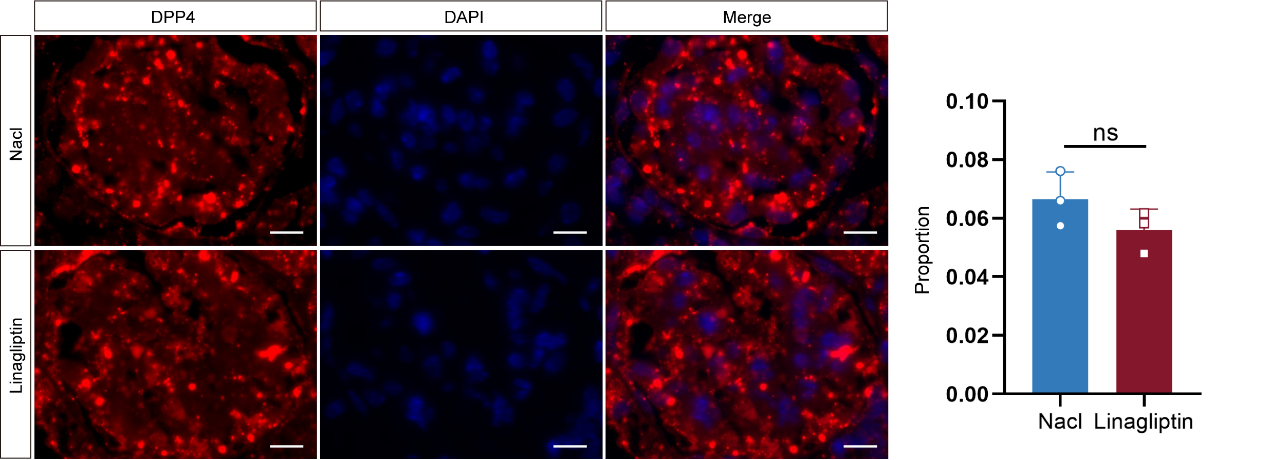


**Figure S3. The expression of DPP4 on glomeruli was determined by immunohistochemistry.**

The expression of DPP4 in glomeruli of the control group and the treatment group was detected by immunohistochemistry. Scale bar = 10 μm, n = 3 for each group.

**Supplementary tables：**

| Si_CXCL12 | | |
| --- | --- | --- |
| Product identification number | Product name | Target sequence |
| siG14417143518 | si-m-Cxcl12-001 | TCTGCATCAGTGACGGTAA |
| siG14417143527 | si-m-Cxcl12-002 | GCACGGCTGAAGAACAACA |
| siG14417143535 | si-m-Cxcl12-003 | GCATTGACCCGAAATTAAA |

**Table S1：Sequence of siRNA_CXCL12.**

| Primer Sequences | |
| --- | --- |
| Forward primer 1 | TGACGGTAAACCAGTCAGCC |
| Reverse primer 1 | CGTGCAACAATCTGAAGGGC |

**Table S2：Sequence of Primer.**
